# Antioxidant supplementation mitigates DNA damage in boar (*Sus scrofa domesticus*) spermatozoa induced by tropical summer

**DOI:** 10.1371/journal.pone.0216143

**Published:** 2019-04-30

**Authors:** Santiago T. Peña, Bruce Gummow, Anthony J. Parker, Damien B. B. P. Paris

**Affiliations:** 1 Gamete and Embryology (GAME) Laboratory, College of Public Health, Medical & Veterinary Sciences, James Cook University, Townsville, Queensland, Australia; 2 Discipline of Veterinary Science, College of Public Health, Medical & Veterinary Sciences, James Cook University, Townsville, Queensland, Australia; 3 Faculty of Veterinary Science, University of Pretoria, Onderstepoort, South Africa; 4 College of Food, Agricultural & Environmental Sciences, Ohio State University, Wooster, Ohio, United States of America; 5 Centre for Tropical Environmental & Sustainability Science, James Cook University, Townsville, Queensland, Australia; Universite Clermont Auvergne, FRANCE

## Abstract

Heat stress-induced sperm DNA damage has recently been demonstrated in boars during tropical summer; which could negatively impact early embryo survival and litter size in sows. Given the boar’s inefficient capacity to sweat, non-pendulous scrotum and low antioxidant activity in seminal plasma, elevated endogenous levels of antioxidants are needed to combat reactive oxygen species induced during periods of heat stress. This should prevent the build-up of pathological levels of DNA damage in boar spermatozoa. Our aim was to investigate whether a combined antioxidant supplement could mitigate sperm DNA damage in boars exposed to tropical summer conditions. Terminal deoxynucleotidyl transferase dUTP nick end labelling and flow cytometry of 20,000 spermatozoa/boar/treatment revealed that boar diets supplemented with 100 g/day custom-mixed antioxidant during peak wet summer effectively reduced sperm DNA damage by as much as 55% after 42 and 84 days treatment respectively (16.1 ± 4.9 peak wet control *vs*. 9.9 ± 4.5 42 day *vs*. 7.2 ± 1.6% 84 day treatments; *P* ≤ 0.05). Supplementation did not improve sperm concentration beyond control levels for either season (*P* > 0.05); nor alter total motility, progressive motility or several other motion parameters measured by computer assisted sperm analysis of 20 x 10^6^ sperm/mL at 38°C (*P* > 0.05). Antioxidant supplementation during tropical summer appears to mitigate the negative impact of heat stress on DNA integrity but not concentration nor motility of boar spermatozoa; which may provide one solution to the problem of summer infertility in the pig.

## Introduction

Tropical countries such as Brazil, Vietnam, The Philippines and Mexico are among the top 10 pork producers globally [1]. Pig production during summer in the tropics can be impacted considerably by the phenomenon of seasonal or summer infertility. Summer temperature and humidity can predispose pigs to heat stress when ambient temperatures rise beyond the animal’s thermal comfort zone at about 18–20° C [2, 3]. This consequently affects food and water consumption, general comfort and reproductive performance, causing significant reduction in profitability. In pigs, poor reproductive performance due to summer infertility has been associated with reduced expression of oestrus and increased pregnancy failure in females [4, 5], and decreased breeding efficiency in males [6, 7].

While the sow plays a central role in overall reproductive success, the inefficient capacity to sweat, non-pendulous scrotum, and the high susceptibility of spermatozoa to temperature shock [8–11], makes the boar particularly vulnerable to the effects of heat stress. Moreover, ambient temperatures above 29°C causes impaired spermatogenesis in Large White boars [12]. Overall, fertility of heat stressed boars is known to be affected by multi-faceted declines in sperm concentration [13], motility and morphology [14, 15], testosterone production [16], ejaculate volume [13] and libido [17].

The relatively high unsaturated fatty acids in the plasma membrane [18] and low antioxidant activity of seminal plasma [19], all contribute to boar sperm’s high sensitivity to peroxidative stress (free radical-mediated oxidative deterioration of polyunsaturated lipids) which can lead to sperm DNA damage during periods of heat stress [20]. Studies in mice show that heat stress induces sperm DNA damage, leading to arrested embryo development and ultimately foetal loss [21]. Our group has recently demonstrated that tropical summer induces 16% DNA damage and reduces concentration of boar spermatozoa without depressing motility [22]. Sperm with greater than 6% DNA fragmentation results in decreased farrowing rates [23]; and, in another study, reduced litter size when sperm DNA fragmentation was greater than 2.1% [24]. Thus, heat stress-induced DNA damaged boar spermatozoa may contribute significantly to early embryo loss in sows.

Antioxidants are substances that inhibit oxidation and ultimately cell damage by neutralising free radicals [25]. Antioxidant supplementation is a common practice geared towards combating oxidative stress and optimising the overall health conditions of many animals but more so particularly in commercial animal production when the demands for growth and reproduction are high [26–29]. In boars specifically, several antioxidants have been identified that improve various sperm quality parameters including Vitamin C [30–32], zinc [33], selenium and Vitamin E [34–36], glutathione [37], and garlic powder [38] among others. Nevertheless, there appears to be no substantial reports demonstrating the benefit of antioxidant supplementation on boar sperm DNA integrity *in vivo*; although one *in vitro* experimental study in which the antioxidant was directly added to the semen extender has been described [39]. In humans, oral administration of 1 g vitamin C and 1 g vitamin E daily for two months [40] or a cocktail of various antioxidants for three months [41], has resulted in improved sperm DNA integrity in men with unexplained infertility and elevated levels of sperm DNA damage. By contrast, another study demonstrated decondensation of sperm DNA after antioxidant supplementation, making it vulnerable to damage, ultimately causing a negative impact on male fertility [42].

Exogenous antioxidant supplementation has been used previously in commercial piggeries to improve overall productivity. In the boar, antioxidants have been shown to improve sperm motility, sperm membrane lipid architecture, mitochondrial membrane potential, viability, survivability and storage, acrosome integrity and functional status, among others [33, 43–45]. While other studies conclude that antioxidants provide little or no value to boar sperm health [34]. Conclusive evidence regarding the effectiveness of antioxidant supplementation to protect boar sperm DNA integrity are limited or at times conflicting; and appear to be related to the specific antioxidant and dosage used, or boar-specific factors [39, 46]. Supplementing anti-lipid peroxidases to thawing and incubation media of frozen-thawed boar spermatozoa protects against DNA fragmentation [47], while the opposite occurs in the presence of glutathione [48]. Nevertheless, improvements in sperm DNA after antioxidant supplementation has been demonstrated in other species such as cattle [49], cats [50] and humans [40, 41]. More specifically, 3 months ingestion of a commercial oral multi-antioxidant supplement comprised of folic acid, zinc, selenium, Vitamins C and E, and garlic resulted in improved sperm DNA integrity, protamine packaging and reduction in seminal reactive oxygen species (ROS) production in infertile men [41]. Such a cocktail of antioxidants are known to either directly neutralize ROS and/or bolster sperm DNA synthesis and protamine packaging [51–54]. To date however, there are no substantial reports validating the potential benefits of antioxidant supplementation on boar sperm DNA integrity. Moreover, it is known that heat stress is associated with reduced expression of oxidative stress-induced antioxidants [55]. As such, we hypothesize that a multi-antioxidant supplement might act synergistically to bolster boar sperm DNA more effectively during periods of heat stress. Therefore, the aim of this study was to investigate whether a combined antioxidant supplement could mitigate sperm DNA damage in boars exposed to tropical summer conditions.

## Materials and methods

### Boars and location

Five Large White boars between 3–3.5 years of age were housed and maintained in an open, gable roof-type facility within individual 3 x 3 metre pens at the College of Public Health, Medical and Veterinary Sciences, James Cook University, Townsville, Queensland, Australia (19°19'46.4"S, 146°45'40.3"E). For inclusion in the study, boars must have met the following minimum standards: having spermatozoa of at least 70% total motility, 65% normal morphology and an ejaculate volume of at least 100 mL. Boars were exposed to prevailing winds and ambient temperatures throughout the day. Each boar was fed 2.3–2.8 kg/day of a commercial pelleted diet (Barastoc, Ridley AgriProducts, Victoria, Australia) to maintain a body score between 3–3.5. Water was provided *ad libitum* via an automatic pig nipple waterer. Experiments were approved by the James Cook University Animal Ethics Committee.

### Climate data

Temperature and relative humidity in Townsville spanning the 42-day period immediately before semen was collected were obtained from the Australian Bureau of Meteorology. This period corresponds to approximately one complete cycle of spermatogenesis in this species [56, 57], during which boars where exposed to ambient environmental conditions. Townsville’s weather, climatic conditions and the procedures by which values for temperature, humidity and temperature-humidity index (THI) were generated were as previously described [22].

### Antioxidant supplementation

Boars were fed 100 g per boar per day custom-mixed multi-antioxidant supplement (PG581 JCU) for 42 and 84 days respectively during the peak wet (hot and wet; January to April 2016) and early dry (cool and dry; May to August 2016) seasons, and semen samples collected and compared to those from the same boars exposed to the peak wet and early dry seasons of the previous year without supplement (February and end of May 2015 respectively). One boar was excluded from the study in the early dry season during the 42-day treatment and a second during the 84-day treatment due to illness. The antioxidant (PG581 JCU) was mixed by a commercial animal feed manufacturer (Rabar Pty Ltd, Queensland, Australia) and contained multiple ingredients including Vitamin E, Vitamin C, Folic acid, β-carotene, Zinc, Selenium, Garlic powder and pollard (as a carrier; Table 1). The ingredients of the antioxidant supplement were based on previous studies showing relevant improvements in the quality of boar or human sperm after supplementation [33, 35, 41, 43–45]. At the time of feeding, 100 g of antioxidant was thoroughly mixed into the first half of the basal feed and given to each boar. The second half of the basal ration was given once the boar had fully consumed the first half to ensure the full antioxidant dose was taken each day.

**Table 1:** Composition of custom-made antioxidant supplement PG581 JCU.

Ingredient	Active level in premix (mg/kg)
Vitamin E	3,250
Vitamin C	25,000
Folic Acid	330
β-carotene	2,250
Zinc^†^	250
Selenium	6
Garlic Powder	75,000
Pollard	*

* acts as carrier

^†^ as zinc sulphate preparation

### Semen collection and processing

At the end of each treatment and from controls, semen was collected from the same n = 5 boars using a dummy sow (Minitube, USA) and gloved hand technique [58]. Briefly, the boar’s penis was directed into a plastic semen collection bag fitted inside a collection cup and covered with non-woven tissue filters (all Minitube, Victoria, Australia) to remove the gel fraction. The collection bag was then placed inside an insulated container containing 38°C water and immediately brought to the laboratory for processing. Raw semen from each boar was diluted 1:3 with 38°C pre-warmed Beltsville Thawing Solution (BTS; pH 7.2 [22, 59]). All reagents were sourced from Sigma-Aldrich (Sydney, New South Wales, Australia), unless otherwise stated. One aliquot was evaluated for sperm concentration using a Neubauer haemocytometer, using standard protocols [60], a second aliquot adjusted to 20 x 10^6^ sperm/mL in BTS for evaluation of sperm motility characteristics using a computer-assisted sperm analyser (CASA; IVOS version 10, Hamilton Thorne Research, Beverly, MA, USA), and a third aliquot evaluated for DNA damage.

### Determination of motility characteristics by CASA

Motility and sperm head characteristics were derived from at least 200 spermatozoa across five random fields. This was achieved by loading each chamber of 38°C pre-warmed Leja Standard Count 4 Chamber Slides (Leja Products, Nieuw-Vennep, Netherlands) with 3 μL of 20 x 10^6^ sperm/mL semen in BTS as previously described [61]. The CASA software was calibrated to the following settings: analysis set-up #7: BOAR; frames acquired, 40/sec; frame rate, 50 Hz; minimum contrast, 60%; minimum cell size, two pixels; minimum static contrast, 30%; straightness threshold, 71.4%; low average-path velocity (VAP) cut-off, 5.0 μm/sec; medium VAP cut-off, 22.0 μm/sec; low straight-line velocity (VSL) cut-off, 11.0 μm/sec; head size (non-motile), two pixels; head intensity (non-motile), 70 pixels; static head size, 0.10–10.0 pixels; static head intensity, 0.10–0.95 pixels; static elongation, 0–60; count slow cells as motile, YES; magnification, 3.20; video source, camera; video frequency, 50; brightfield, NO; illumination intensity, 2381 and temperature, 38°C. The following characteristics were evaluated: total motility, progressive motility of the whole sample, average-path velocity (VAP; μm/sec), straight-line velocity (VSL; μm/sec), curvilinear velocity (VCL; μm/sec), amplitude of lateral head displacement (ALH; μm), beat cross frequency (BCF; Hz), straightness (STR; ratio of VSL/VAP), linearity (LIN; ratio of VSL/VCL) and elongation (ELO; ratio in % of head width to head length) as previously described [61, 62].

### Sperm DNA integrity assay and flow cytometry analysis

The procedures used for sperm DNA integrity analysis were as described by Peña *et al*. [22]. Briefly, BTS-diluted semen samples were purified by Percoll gradient centrifugation to remove seminal plasma and possibly dead and damaged spermatozoa [63]. The final sperm pellet was adjusted to 5 x 10^6^ sperm/mL in BTS. Boar spermatozoa was stained using the Terminal deoxynucleotidyl transferase dUTP nick end labelling assay according to manufacturer’s instructions (TUNEL; *In Situ* Cell Death Detection Kit, Fluorescein, Version 17, Nov 2012, Roche Diagnostics, Mannheim, Germany) with modifications. Six control samples (2 positive, 2 negative, and 2 unlabelled) were prepared in parallel using pooled semen. These were used to accurately gate different populations of spermatozoa in the flow cytometer before experimental samples were analysed as previously described [22]. The TUNEL reaction labels DNA damaged cells positive for Fluorescein isothiocyanate (FITC). Positive controls (P1 and P2) and all test samples were incubated in 50 μL TUNEL reaction mixture containing enzyme while the Negative controls (N1 and N2) were incubated in TUNEL labelling solution without the enzyme. Unlabelled controls (U1 and U2) were incubated in PBS. Moreover, U2, N2, P2 and all test samples were subsequently incubated with 5 μg/mL of the nucleic acid stain 4', 6-diamidino-2-phenylindole (DAPI) in PBS for 20 min at room temperature to ensure that only nucleated TUNEL-positive spermatozoa were accounted for as DNA damaged cells during analysis by FACS. The specificity of sperm staining was validated using fluorescent microscopy [22], and showed FITC/DAPI positive DNA damaged sperm heads in green alongside DAPI positive DNA intact boar sperm heads in blue.

All samples were evaluated using a CyanADP flow cytometer (Dako Cytomation, Glostrup, Denmark). Spermatozoa were identified by their forward and side scatter profiles using a scatter-area *vs*. scatter-height gate previously calibrated specifically for boar spermatozoa. Data were analysed using Summit 4.3 software (Dako Cytomation). The flow cytometer was set to analyse 20,000 cells per sample at about 150 events/sec. Prior to evaluating test samples, control samples were used to accurately define the different cell staining populations delineated into four distinct quadrants by adjusting both vertical and horizontal thresholds: (i) R3, FITC-positive cells only; (ii) R4, both FITC and DAPI-positive cells; (iii) R5, unstained cells; and (iv) R6, DAPI-positive cells only [22]. Sample N2 (Negative control in Label Solution with DAPI) was used to set a 0.5% threshold cut-off before running all test samples. Cells in R4 were designated as nucleated DNA damaged spermatozoa, expressed as a percentage of the total number of cells analysed within the gated area.

### Data presentation and statistical analyses

Standard tests to check for normality and variance in the data were performed using the Shapiro-Wilk test and Levene’s test, respectively and data were transformed using Log_10_ where necessary before any statistical analysis was done. Differences in test parameters were analysed using the parametric paired sample tests (sperm DNA damage, sperm concentration and most CASA parameters) or independent sample T-tests (involving the 42 and 84 days antioxidant supplementation in winter) in SPSS (SPSS Statistics version 22, IBM Corporation, NY, USA). Where a parametric test was inappropriate (i.e. assumptions for parametric tests were not met), a 2-sample related test (mean maximum, mean minimum and daily mean temperatures, humidity and THI values) or Mann-Witney test (CASA parameters for VSL and ALH) was used to determine if values were significantly different (*P* ≤ 0.05).

## Results

Daily mean temperatures spanning the 42-day period immediately prior to semen collection were consistently hotter during peak wet than early dry season (*P* ≤ 0.05, Table 2). Moreover, daily mean temperatures were identical for the control and 42-day supplement groups during either the peak wet or early dry seasons. Daily mean relative humidity was generally similar for most treatments, ranging from 70–73%. However, the 84-day supplement group during the peak wet was more humid while the early dry control was dryer. Daily mean temperature-humidity index was consistently higher during the peak wet than early dry season (*P* ≤ 0.05), although values started to decline in the 84-day supplement groups during the peak wet, but was lowest for the early dry season (*P* ≤ 0.05).

**Table 2:** Mean (± SEM) ambient temperature, relative humidity and temperature-humidity index in Townsville, North Queensland, Australia spanning the 42 day treatment period immediately preceding semen collection during the peak wet and early dry seasons.

	Peak Wet Control (Feb 2015)	Peak Wet + 42 day Antiox (Feb 2016)	Peak Wet + 84 day Antiox (Apr 2016)	Early Dry Control (May 2015)	Early Dry + 42 day Antiox (Jun 2016)	Early Dry + 84 day Antiox (Aug 2016)
**Ambient Temperature (°C)**						
Daily Mean	29.2 ± 0.2^a^	29.3 ± 0.2^a^	27.3 ± 0.2^b^	24.2 ± 0.4^c^	23.7 ± 0.3^c^	21.1 ± 0.3^d^
**Relative Humidity (%)**						
Daily Mean	71.4 ± 1.2^bc^	72.4 ± 1.0^bc^	77.1 ± 1.3^a^	61.9 ± 2.1^d^	73.0 ± 1.4^ab^	70.0 ± 2.3^c^
**Temperature-Humidity Index (THI)**						
Daily Mean	92.9 ± 1.1^a^	93.4 ± 1.2^a^	86.3 ± 0.7^b^	75.8 ± 0.9^c^	75.5 ± 0.6^c^	70.2 ± 0.7^d^

Different letters indicate a significant difference between treatments (*P* ≤ 0.05).

**Fig 1:**
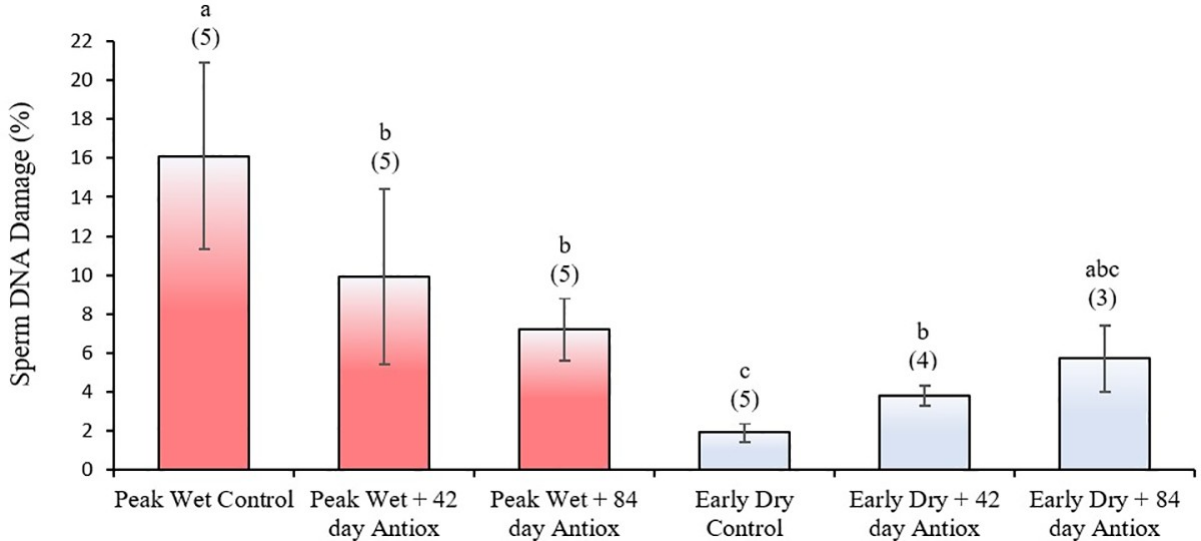
Mean (± SEM) percentage of DNA damage in boar spermatozoa collected after no (control), 42 or 84 days antioxidant supplementation during peak wet and early dry seasons. Different letters indicate significant difference between treatment groups (*P* ≤ 0.05); numbers in parenthesis indicate sample size.

Antioxidant supplementation of boars during the peak wet resulted in more than a 1.6 and 2.2-fold reduction of DNA-damaged spermatozoa after both 42 and 84 days treatment, respectively (*P* ≤ 0.05; Fig 1). Peak wet supplementation did not reduce DNA damage to basal levels observed during the early dry season, but values were similar to those observed during supplementation in the early dry.

**Fig 2:**
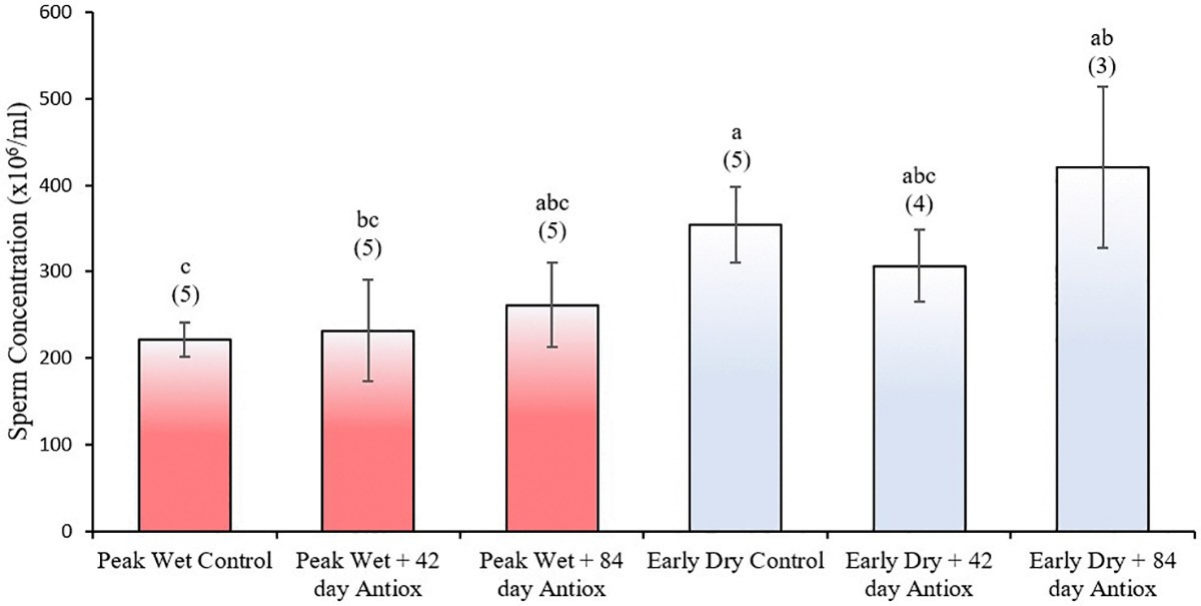
Mean (± SEM) concentration of boar spermatozoa collected after no (control), 42 or 84 days antioxidant supplementation during peak wet and early dry seasons. Different letters indicate a significant difference between treatment groups (*P* ≤ 0.05); numbers in parenthesis indicate sample size.

While sperm concentration was lower in the peak wet compared to early dry control (*P* ≤ 0.05; Fig 2), antioxidant supplementation did not improve sperm concentration beyond control levels for either season (*P* > 0.05).

**Fig 3:**
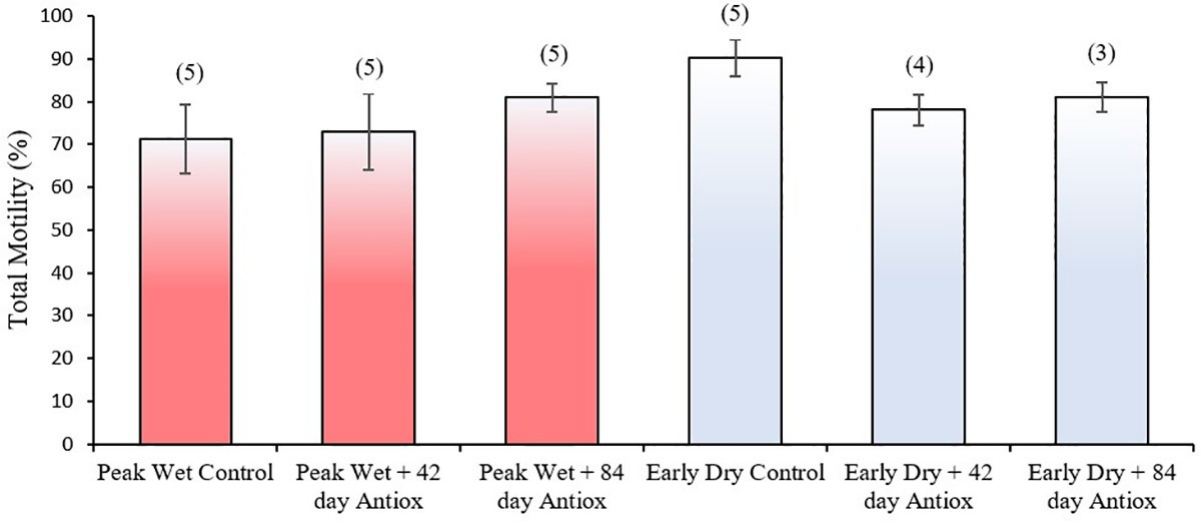
Mean (± SEM) percentage of total motility of boar spermatozoa collected after no (control), 42 or 84 days antioxidant supplementation during peak wet and early dry seasons. No significant difference between treatment groups (*P* > 0.05); numbers in parenthesis indicate sample size.

**Fig 4:**
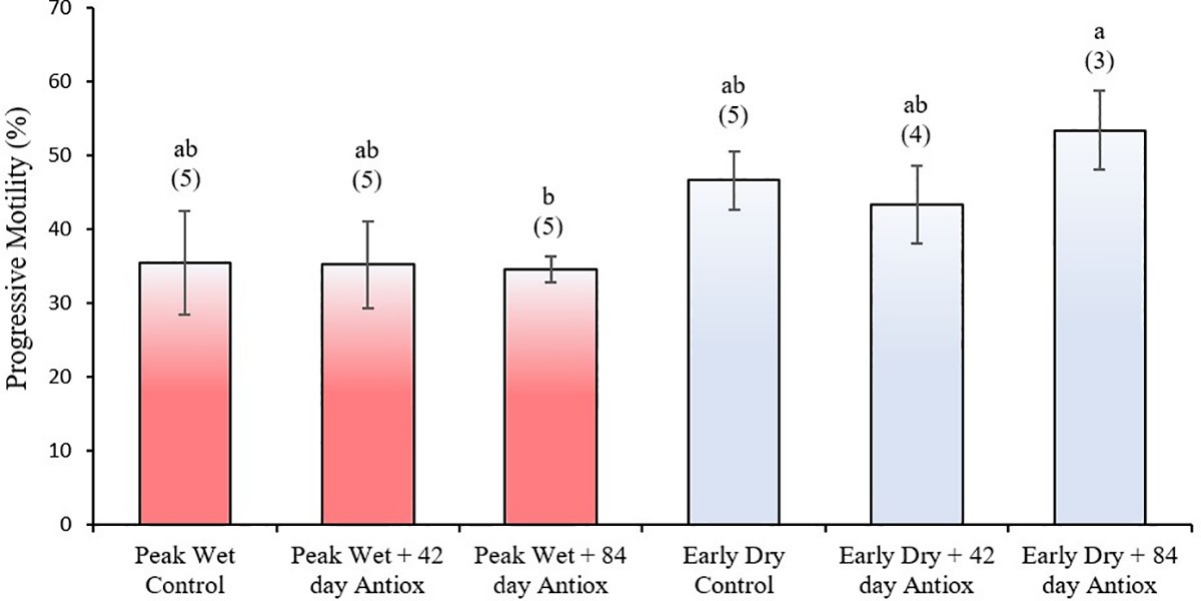
Mean (± SEM) percentage of progressively motile boar spermatozoa collected after no (control), 42 or 84 days antioxidant supplementation during peak wet and early dry seasons. Different letters indicate a significant difference between treatment groups (*P* ≤ 0.05); numbers in parenthesis indicate sample size.

Total sperm motility was similar in the peak wet and early dry and this was not altered by 42 or 84-day treatment with antioxidants during either season (*P* > 0.05; Fig 3). Similarly, the number of progressively motile spermatozoa were similar in the peak wet and early dry and this was not altered by 42 or 84 day treatment with antioxidants during either season (*P* ≥ 0.05; Fig 4). However, there were more progressively motile spermatozoa after 84 days antioxidant supplementation during early dry than peak wet season (*P* ≤ 0.05).

Detailed sperm motility and head shape characteristics determined by CASA are shown in Table 3. Average path velocity, straight-line velocity, curvilinear velocity, amplitude of lateral head displacement and beat cross frequency were similar in the peak wet and early dry and this was not altered by 42 or 84-day treatment with antioxidants during either season (*P* > 0.05). Sperm elongation was higher after 42 days antioxidant supplementation in the early dry but also after 84 days treatment in both early dry and peak wet seasons, respectively (*P* ≤ 0.05). Straightness and linearity of spermatozoa only increased compared to control after 84 days supplementation during the early dry season (*P* ≤ 0.05).

**Table 3:** Mean (± SEM) sperm motility and head shape characteristics in boar ejaculates collected after no (control), 42 days or 84 days antioxidant supplementation during peak wet and early dry seasons in Townsville, North Queensland, Australia.

CASA Parameters	Peak Wet Control (n = 5)	Peak Wet + 42 day Antiox (n = 5)	Peak Wet + 84 day Antiox (n = 5)	Early Dry Control (n = 5)	Early Dry + 42 day Antiox (n = 4)	Early Dry + 84 day Antiox (n = 3)
VAP	26.7 ± 2.7	31.9 ± 2.7	32.5 ± 2.7	38.8 ± 4.5	33.8 ± 1.7	35.6 ± 2.1
VSL	22.2 ± 2.4	25.8 ± 2.5	26.8 ± 2.5	30.7 ± 3.5	28.9 ± 1.2	31.3 ± 2.1
VCL	45.9 ± 4.1	55.9 ± 4.5	52.7 ± 3.6	68.3 ± 7.0	56.2 ± 2.3	59.0 ± 2.3
ALH	2.3 ± 0.2	2.7 ± 0.2	2.5 ± 0.2	3.4 ± 0.3	2.7 ± 0.1	2.8 ± 0.1
BCF	21.1 ± 0.6	17.3 ± 0.6	16.9 ± 1.2	19.1 ± 1.5	18.3 ± 1.2	20.2 ± 1.9
STR	76.9 ± 2.2^ab^	76.1 ± 2.5^ab^	76.4 ± 1.2^b^	74.1 ± 1.3^b^	80.6 ± 2.1^ab^	83.2 ± 2.8^a^
LIN	47.3 ± 2.1^ab^	46.4 ± 2.7^ab^	47.9 ± 1.6^ab^	44.8 ± 1.2^b^	51.2 ± 3.0^ab^	52.0 ± 3.1^a^
ELONG	80.3 ± 1.2^b^	86.9 ± 3.1^ab^	87.7 ± 2.3^a^	78.3 ± 1.3^b^	87.8 ± 1.0^a^	88.0 ± 0.7^a^

Different letters indicate a significant difference between treatment groups (*P* ≤ 0.05).VAP, average-path velocity (μm/sec); VSL, straight-line velocity (μm/sec); VCL, curvilinear velocity (μm/sec); ALH, amplitude of lateral head displacement (μm); BCF, beat cross frequency (Hz); STR, straightness (ratio of VSL/VAP); LIN, linearity (ratio of VSL/VCL); ELONG, elongation (ratio in % of head width to head length).

## Discussion

The negative impact of heat stress on sperm DNA integrity coupled with its downstream effect on early embryo development [20], presents a new challenge to maintaining seasonal sperm quality in boars [39, 64]. Here, we demonstrate for the first time the beneficial effect of a multi-antioxidant supplement in reducing DNA damage in boar spermatozoa during periods of tropical heat stress. Supplementation of boars at 100 g/day using a custom-made antioxidant formula resulted in 38% to more than 55% reduction in sperm DNA damage after 42 and 84 days, respectively.

Baseline levels of sperm DNA damage occur naturally in the final stages of spermiogenesis [65]. Physiologically, it helps to relieve torsional stress during the DNA packaging process into the compact nucleus of the sperm head [65]. For example, our study has shown that the baseline level of sperm DNA damage in boars raised under tropical conditions during the early dry (when environmental temperature is cool) is about 1%. There are however, several additional causes of sperm DNA damage including environmental stress, toxicants, pollution, infection, poor nutrition and low antioxidant activity in the seminal plasma [65, 66]. Oxidative stress-induced antioxidants are reduced in cells during heat stress [55], predisposing them to DNA attack by reactive oxygen species. Spermatozoa are specifically vulnerable to oxidative damage due their inherent high level of polyunsaturated fatty acids (PUFAs) in the plasma membrane [67, 68]. Excessive production of reactive oxygen species (ROS) increases rates of cellular damage [69], and in sperm increase the rate of sperm ATP depletion; which in turn leads to insufficient axonemal phosphorylation, lipid peroxidation, and loss of motility and viability [70]. As such, tropical heat stress encountered by boars during the peak wet season when the ambient temperature, humidity and THI are high appears to be the major contributor to the substantial DNA strand breakages that occur in boar sperm [22] during this time. Given that spermatozoa lack DNA repair machinery, some could be released from the germinal epithelium still carrying their broken DNA [65]. The female reproductive tract is known to limit the migration of many types of abnormal sperm through natural barriers in the cervix and uterotubal junction [71]. However, Percoll purification often enriches raw semen for fertilization competent spermatozoa [72] to reach and fertilize oocytes. Given we detected over 16% DNA damage during summer in Percoll-purified spermatozoa, suggests that it is this enriched population of spermatozoa most likely to participate in fertilization during natural breeding or artificial insemination, with the potential to adversely affect embryo viability. This is further supported by observations in pigs, mice and humans that show a decrease in litter size or pregnancy rate respectively, when sperm DNA damage increases above species-specific thresholds [23, 24, 73, 74]. As such, it is important to test for sperm DNA integrity in ‘gradient-enriched’ populations of spermatozoa. Nevertheless, results found in our study during periods of heat stress appear to support the role of antioxidants in neutralizing free radical activity and protecting sperm DNA from ROS that are already produced [75].

Our study tested a multi-antioxidant formulation, an approach that can increase the putative synergistic effect each compound has on sperm quality, as observed in other studies using a mixed formula [68, 76, 77]. Our antioxidant formula given at 100 g/day resulted in a 1.6 to 2.2-fold reduction in sperm DNA damage after 42 and 84 days, respectively. While the beneficial compound(s) and mechanism by which this antioxidant cocktail functions in protecting sperm DNA is still unclear, the reduction in sperm DNA damage can be related to other positive effects of antioxidants in boar sperm biology. Selenium, a crucial component in swine nutrition, serves as a raw material in the synthesis of selenoprotein. Selenoprotein plays a significant role in antioxidant system regulation in the body [54], from which a popular Se-dependent enzyme glutathione peroxidase (GSH-Px) depends. Glutathione and vitamin E increase sperm production but also protect against lipid peroxidation [78]. In fact, lipid peroxidation, as measured by the levels of ascorbate-induced thiobarbituric acid reactive substances (TBARS), was inhibited by as much as 62% and 57% using water-soluble vitamin E analog (TROLOX) and GSH, respectively [78]. Moreover, garlic, which is also part of our antioxidant cocktail, is able to regulate leukocyte cell proliferation and cytokine production [52] and this anti-inflammatory effect could potentially reduce ROS production by seminal leukocytes.

Where pigs are reared in groups/herds, administration of a multi-antioxidant supplement via their feed is both convenient and has been shown to have synergistic effects. For example, selenium and Vitamin E tend to produce better results in improving boar sperm motility, concentration and/or morphology when given together [35]. Similarly, Vitamin B12 and folic acids tend to produce better results on folate and homocysteine metabolism in pigs during early pregnancy [79]. Overall, our work and the above studies suggest a cocktail of antioxidants in a supplement formula appears to be more beneficial than a single antioxidant approach to treating boars.

Nevertheless, not all antioxidants are guaranteed to protect boar sperm against DNA damage. While survival of boar sperm improved, adding magnesium fumarate to Biosolwens extender increased the proportion of sperm DNA damage [39]. Moreover, zinc in the form of zinc-methionate at 200 ppm adversely affected boar sperm quality including increased sperm DNA damage [80]. It is not known whether antioxidant supplementation in our study has led to accumulated levels of zinc in the testis or spermatozoa of our boars, but in our case zinc was administered as zinc sulphate at a recommended dose of ~100 ppm [33]. Zinc is known to facilitate the condensation of DNA protamine 2 [51], however, one study reported decondensation of sperm DNA after zinc and selenium supplementation, making it vulnerable to damage [42]. Coincidentally, we observed a significant increase in sperm head width (via the elongation parameter) in nearly all antioxidant treatments irrespective of season (Table 3). This may reflect impaired DNA compaction and possibly low-level strand breaks associated with these antioxidants in spermatozoa obtained from our treatment boars. Perhaps this might partly explain the increase in sperm DNA damage compared to control after 42 days treatment during the much cooler early dry season (Fig 1). Given these levels were similar to those observed in antioxidant treated groups during the peak wet but both were significantly lower than control at this time, suggests zinc may be a beneficial antioxidant during periods of tropical heat stress but may be detrimental as a long-term general supplement.

Interestingly, despite sperm concentration in the peak wet control being significantly lower that the early dry control, we did not observe any significant improvement in sperm concentration nor sperm motility after antioxidant supplementation. Some previous studies also showed no improvement in sperm motility [41, 43], and selenium has been reported to reduce sperm motility *in vitro* when added to extender [76]. However, in other studies [32, 35, 36, 38] improved sperm motility, concentration and/or morphology were the primary consequences of antioxidant supplementation; with one paper specifically highlighting the beneficial effect of antioxidants Selenium and Vitamin E during the warm season [35]. These papers were the basis upon which we selected compounds for inclusion in our antioxidant formula. However, the mechanisms by which antioxidants support DNA structural integrity is still not clear and may not necessarily be linked to pathways that enhance sperm motility and increased spermatogenesis during periods of heat stress. Our previous study showed that tropical heat stress does not affect sperm motility in boars [22], suggesting more detailed studies are needed on the mechanism by which heat stress acts on sperm physiology and the protective role antioxidants play across the different sperm quality parameters.

In conclusion, antioxidant supplementation appears to be an effective measure to mitigate the negative impact of heat stress on sperm DNA integrity but not sperm concentration nor motility during tropical summer. While further research is needed to identify which specific antioxidant(s) in the formula confer this DNA protection and their precise mechanism of action, our study provides a practical solution to improving boar fertility during periods of heat stress, which may greatly improve pig production during summer in tropical and sub-tropical environments.
